# Methane release from the southern Brazilian margin during the last glacial

**DOI:** 10.1038/s41598-018-24420-0

**Published:** 2018-04-13

**Authors:** R. C. Portilho-Ramos, A. P. S. Cruz, C. F. Barbosa, A. E. Rathburn, S. Mulitza, I. M. Venancio, T. Schwenk, C. Rühlemann, L. Vidal, C. M. Chiessi, C. S. Silveira

**Affiliations:** 1 MARUM - Center for Marine Environmental Sciences, University of Bremen, Leobener Strasse, 28359 Bremen, Germany; 2 Programa de Pós Graduação em Geoquímica, Universidade Federal Fluminense, Outeiro São João Batista S/N, 24020-141 Niterói RJ, Brazil; 3 Institute of Geosciences, University of São Paulo, Rua do Lago 562, 05508-080 São Paulo SP, Brazil; 4 Department of Geological Sciences, California State University Bakersfield, 9001 Stockdale Highway, Bakersfield, CA USA; 5 Center for Weather Forecasting and Climate Studies (CPTEC), National Institute for Space Research (INPE), Cachoeira Paulista, Brazil; 6 BGR – Federal Institute for Geosciences and Natural Resources, Stilleweg 2, 30655 Hannover, Germany; 7 Aix-Marseille University, CNRS, IRD, CEREGE UM34, 13545 Aix-en-Provence, France; 8 School of Arts, Sciences and Humanities, University of São Paulo, Av. Arlindo Béttio 1000, 03828-000 São Paulo SP, Brazil

## Abstract

Seafloor methane release can significantly affect the global carbon cycle and climate. Appreciable quantities of methane are stored in continental margin sediments as shallow gas and hydrate deposits, and changes in pressure, temperature and/or bottom-currents can liberate significant amounts of this greenhouse gas. Understanding the spatial and temporal dynamics of marine methane deposits and their relationships to environmental change are critical for assessing past and future carbon cycle and climate change. Here we present foraminiferal stable carbon isotope and sediment mineralogy records suggesting for the first time that seafloor methane release occurred along the southern Brazilian margin during the last glacial period (40–20 cal ka BP). Our results show that shallow gas deposits on the southern Brazilian margin responded to glacial−interglacial paleoceanographic changes releasing methane due to the synergy of sea level lowstand, warmer bottom waters and vigorous bottom currents during the last glacial period. High sea level during the Holocene resulted in an upslope shift of the Brazil Current, cooling the bottom waters and reducing bottom current strength, reducing methane emissions from the southern Brazilian margin.

## Introduction

Methane is an important greenhouse gas and alterations in its atmospheric concentration have been associated with changes in global temperature over orbital and millennial time-scales^1^. Appreciable quantities of methane are sequestered in marine sediments in the form of ice-like gas hydrate deposits (ca. 1,600–2,000 Pg of C)^2^ and shallow subsurface gas deposits, which are sensitive to changes in environmental conditions on the seafloor^3–5^. Changes in pressure (*i*.*e*. sea level changes), bottom water temperature and bottom current strength have been the main triggers for seafloor gas methane seepage^6–9^. A decrease in pressure and an increase in bottom water temperature and flow speed have great potential to destabilize gas deposits and release considerable amounts of methane to the ocean^3,10^. An appreciable amount of methane released from seafloor gas deposits is consumed by microorganisms in the water column, potentially altering seawater carbonate chemistry and promoting ocean acidification^11^. A portion of the released methane may reach the atmosphere, and significantly contribute to global warming^2,11^, although the amount of methane that reaches the atmosphere is still debated^12^. An in-depth understanding of the temporal evolution and mechanisms related to seafloor gas methane release are critical for assessing ancient changes in the climate and chemistry of the oceans and the atmosphere, with potential consequences for future global carbon cycle and climate projections^2,11^.

The calcareous shells of benthic foraminifera living in cold seep habitats have more negative stable carbon isotopic (*δ*^13^C) values compared to the same species living in non-seep environments (see supplementary information)^13–15^ because of the incorporation of negative *δ*^13^C from ambient methane^16^. Recent laboratory culture experiments using pressure chambers to expose deep-water foraminifera species to labeled methane showed that these species can live in a methane-laden environment and that the *δ*^13^C of calcareous benthic foraminiferal tests are influenced by the δ^13^C signature of methane^17^. Thus, anomalously negative δ^13^C excursions in fossil foraminiferal carbonate from sedimentary records are used to assess the geologic history of methane seepage^6,7,18–20^. Additionally, tests of planktonic foraminifera deposited in sites influenced by methane seepage may also show negative δ^13^C excursions due to carbonate overgrowth^21^.

The southern Brazilian margin (SBM) is a passive hydrocarbon basin where several circular and elliptical depressions of collapsed sediments (*i*.*e*. large pockmark fields) have been associated with methane release from hydrate reservoirs and shallow free gas layers^5,22,23^. Multi-channel seismic data revealed complex subsurface faults, fractures and channels connecting deep and shallow gas hydrates as well as shallow subsurface free-gas deposits to the pockmarks^5,23^. The presence of chemosynthetic communities^24,25^ and high biogenic methane concentrations^23^ are suggestive of modern active cold methane seeps on the SBM, while the presence of authigenic carbonate nodules with *δ*^13^C values between −16‰ and −30‰ likely resulted from past methane release^26,27^. However, only a few studies have examined methane seepage history in the Brazilian margin^26,27^.

**Figure 1:**
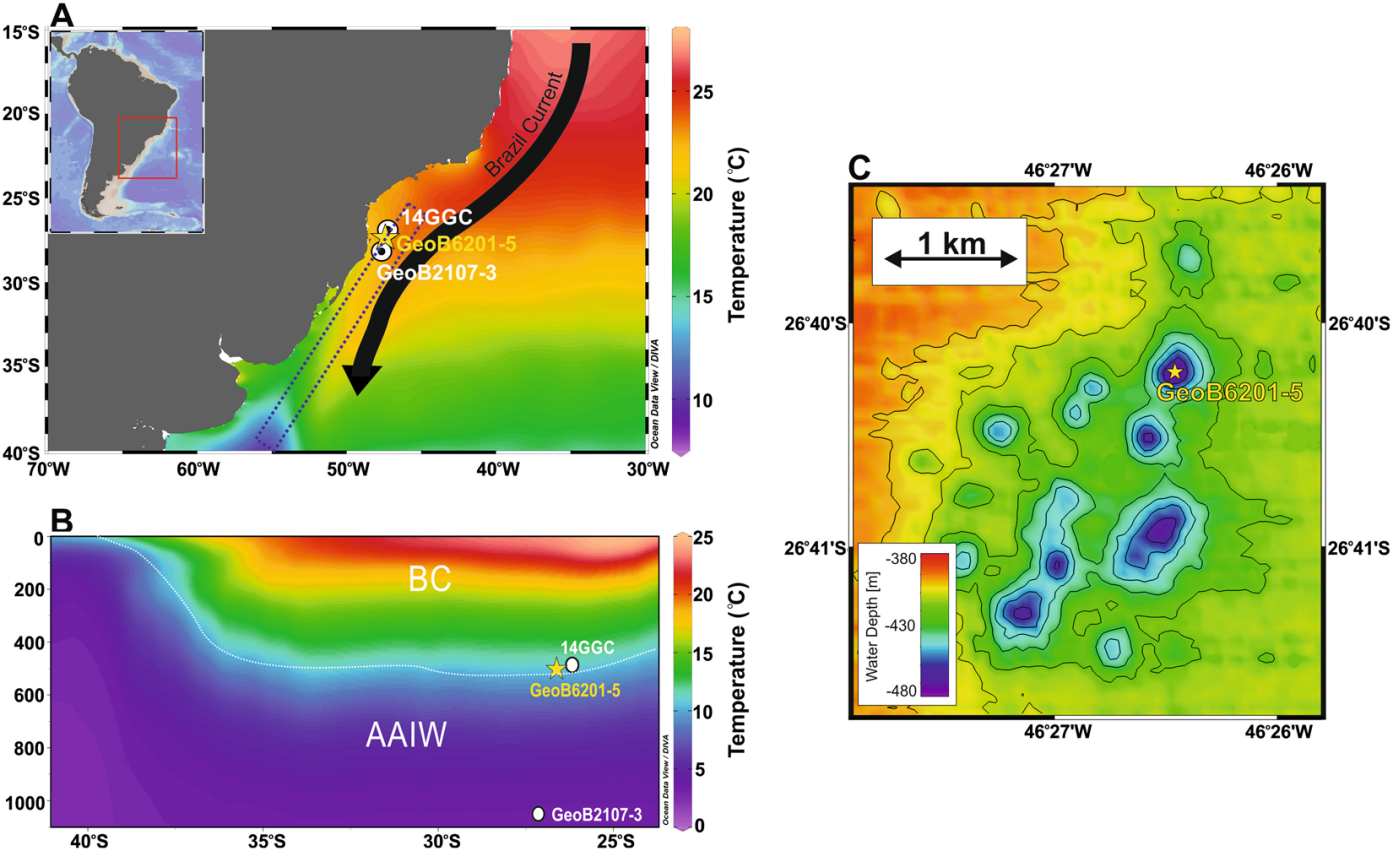
Location of core GeoB6201-5 (this study) and adjacent control cores GeoB2107-3^64^ and 14GGC^30^. (**A**) Sea surface temperature in the western South Atlantic Ocean^65^ showing the southward transport of warm tropical waters by the Brazil Current (red arrow). (**B**) Depth profile (dashed rectangle in A) showing water mass geometry in the western South Atlantic Ocean; Tropical Water (TW), South Atlantic Central Water (SACW) and Antarctic Intermediated Water (AAIW). White dotted line indicated the isopycnal σ = 27.1 marking the boundary between the lighter SACW and the denser AAIW^29,66^. Both TW and SACW are transported southward by the Brazil Current south of 20°S^67^ (**C**) 25m-resolution bathymetric map of the pockmark field at the southern Brazilian Margin showing funnel-shaped depressions. Core GeoB6201-5 was collected from one of these depressions^28^. (**A**) and (**B**) were prepared using Ocean Data View software^68^ (ODV - version, 4.7.9., http://odv.awi.de, 2017). The bathymetric data in **C** were collected with a Hydrosweep DS2 system during the RV Meteor cruise M46/2(CIT). Processing and visualization was done with software Fledermaus Pro 7.7.6 (QPS; http://www.qps.nl). The map represents a grid with 25 m resolution.

To determine the temporal evolution of past seafloor methane seepage at the SBM, we examined the δ^13^C signatures of benthic (*Cibicides spp*. and *Uvigerina* spp., ≥250 μm) and planktonic (*Globigerinoides ruber* and *Globigerinoides sacculifer*, ≥250 μm) foraminifera from marine sediment core GeoB6201-5 (26°40′S, 46°26′W, 475 m water depth, 235 cm long) collected at a funnel-shaped depression (*i*.*e*. pockmark) located on the SBM (Fig. 1 and supplementary Figure S1)^28^. The pockmark is placed at the boundary between two southern sourced water masses, the denser Antarctic Intermediated Water (AAIW) and the less dense South Atlantic Central Water (SACW) that are separated by the 27.1 isopycnal at ca. 500 m water depth^29^ (Fig. 1B; see supplementary information for further details). We compare *δ*^13^C records from core GeoB6201-5 to *δ*^13^C records from nearby cores collected outside of the pockmark, namely GeoB2107-3 (27°17′S, 46°45′W, 1048 m water depth, 780 cm long) and 14GGC (26°68′S, 46°50′W, 441 m water depth, 200 cm long)^30^ (Fig. 1A and supplementary information). The *δ*^13^C records from these two sediment cores provide the regional background conditions not affected by methane seepage. The presence of authigenic carbonate precipitation derived by the anaerobic oxidation of methane (AOM) in core GeoB6201-5 was determined by the elemental composition of individual benthic foraminiferal tests (*Cibicides wuellerstorfi*) as well as the mineralogical composition of sediments (see Material and methods).

The age model of GeoB6201-5 is based on six AMS ^14^C ages performed on planktonic foraminifera *G.* *ruber* (supplementary Table S1), regional planktonic foraminiferal biostratigraphy and stable oxygen isotope (*δ*^18^O) stratigraphy (supplementary Figs. S2, S3 and S4). The age model and associated uncertainties were calculated with the R script BACON version 2.2^31^ with the IntCal13 calibration curve^32^ and a reservoir correction age of 400 ± 100 yr (supplementary Fig. S2). We verified our ^14^C-based age model for the last 40 cal ka BP by confronting the *Cibicides* spp. *δ*^18^O record from GeoB6201-5 to a *Cibicides* spp. *δ*^18^O record from nearby core GeoB2107-3 as well as to a recently published intermediate water South Atlantic benthic *δ*^18^O stack^33^ (supplementary Fig. S2). The chronology of core GeoB6201-5 is additionally supported by planktonic foraminifera biostratigraphy^34,35^, where the presence of *Globorotalia menardii* and the low abundance of *Globorotalia inflata* indicate Biozone Z (Holocene), while the absence of *G*. *menardii* and the high abundance of *G*,*inflata* characterize the glacial Biozone Y (last glacial) (supplementary Fig. S2b). The chronology of core GeoB2107-3 was previously published in Heil^36^ and Gu^37^.

**Figure 2:**
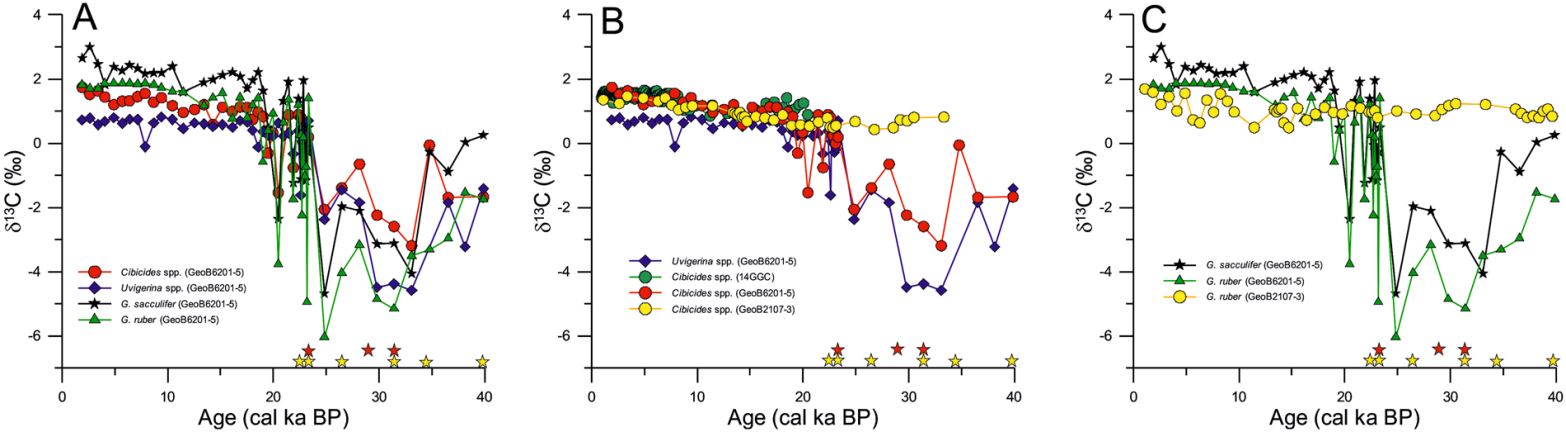
Methane release from southern Brazilian margin during the last glacial period. (**A**) Downcore benthic and planktonic foraminifera *δ*^13^C records from the seep core GeoB6201-5 showing the strong *δ*^13^C depletion between 40 and 20 cal ka BP. (**B**) Comparison of benthic foraminifera *Uvigerina* spp. and *Cibicides* spp. *δ*^13^C records from seep core GeoB6201-5 and adjacent non-seep cores GeoB2107-3 and 14GGC^30^. (**C**) Comparison of planktonic foraminifera *Globigerinoides ruber* and *Globigerinoides sacculifer δ*^13^C records from seep core GeoB6201-5 and adjacent non-seep cores GeoB2107-3. Stars in the bottom indicates depth with presence of High-Mg calcite derived from XRD (Red stars) and presence of Mg derived from SEM-EDX (Yellow stars).

The Holocene *δ*^13^C values of planktonic foraminifera species *G.* *ruber* (1.6‰–1.8‰) and *G*,*sacculifer* (2.2–3.0‰) as well as benthic foraminifera *Cibicides spp*. (1.2–1.7‰) and *Uvigerina* spp,(−0.1–0.8‰) from core GeoB6201-5 are consistent with those from non-seep surface sediments from the western South Atlantic Ocean^38,39^ and with downcore values from adjacent cores GeoB2107-3 and 14GGC^30^ (Fig. 2). On the other hand, anomalous negative *δ*^13^C values in both planktonic (down to −6‰) and benthic (down to −4.6‰) species are present in the last glacial (40–20 cal ka BP) section of core GeoB6201-5 (Fig. 2). These values are significantly more negative than the values of the adjacent non-seep cores GeoB2107-3 and 14GGC^30^.

**Figure 3:**
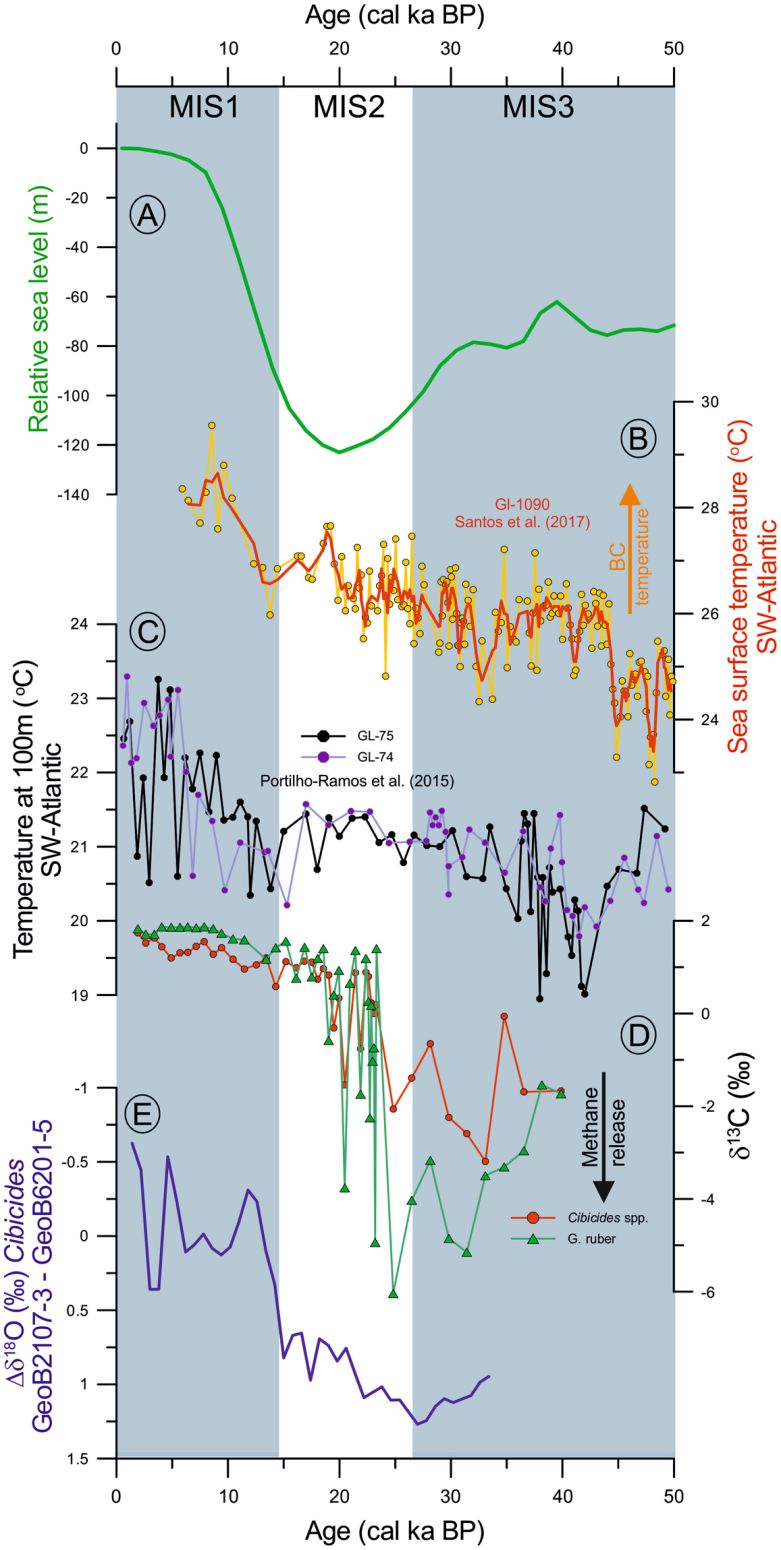
Response of methane release from the southern Brazilian margin to paleoceanographic changes over the last 50 cal ka BP. (**A**) Relative sea level changes over the last 50 cal ka BP^55^. (**B**) Sea Surface temperatures derived from *Globigerinoides ruber* Mg/Ca from core GL-1090^57^. (**C**) Temperature at 100m-water depth estimated using the modern analogue technic based on planktonic foraminifera from cores GL-75 and GL-74^58^. (**D**) Stable carbon isotope (*δ*^13^C) records of *Cibicides* spp. and *Globigerinoides ruber* showing the strong depletion that indicate methane release from the SBM between 40 and 20 cal ka BP (this study). (**E**) Vertical thermal gradient in the southern Brazilian margin derived from the stable oxygen isotopic differences (*δ*^18^O) of *Cibicides* spp. between core GeoB6201-5 (at 475 m water depth) and GeoB2107-3 (1048 m water depth).

The absence of anomalously negative *δ*^13^C values in contemporaneous foraminifera from the adjacent cores (GeoB2107-3 and 14GGC^30^; Fig. 2B–C) rules out the possibility of an enhanced primary productivity event and associated increased carbon flux to the seafloor (the so called Mackensen effect)^40^ as well as the influence of a bottom water mass with significantly lighter *δ*^13^C^30,39^ as the main sources for the observed negative *δ*^13^C excursions recorded in core GeoB6201-5. The anomalously negative benthic foraminiferal *δ*^13^C values in our records are consistent with those previously reported from modern and past cold-methane seeps (supplementary Table S2)^13,14,19,41–43^. For example, living epifaunal specimens of *Cibicidoides wuellerstorfi* from a cold seep in Monterey Bay off California show mean *δ*^13^C values as low as −3.3‰^14^ while the same species from our core GeoB6201-5 had *δ*^13^C values down to −3.2‰ (Fig. 3B). The infaunal species *U*. *peregrina*, from a Blake Ridge seep diapir in the NE-Atlantic had a mean *δ*^13^C of −4.2‰ for the late Holocene^43^ while *U*. *peregrina* specimens from our core GeoB6201-5 had values down to −4.6‰ (Fig. 3B). This suggests that the carbonate tests of foraminifera were precipitated under the influence of low *δ*^13^C values of dissolved inorganic carbon (DIC) due to sulfate-dependent AOM^44–46^.

Depleted-*δ*^13^C signals in foraminifera shells can be also related, in part, to authigenic carbonate overgrowth induced by AOM^20,21,41,47^. Our specimens of planktonic foraminifera also show anomalous negative *δ*^13^C values down to −6‰ (Fig. 2C), which are unlikely to have been caused by AOM in the water column, as suggested in previous studies^6,19^. Instead, gas methane is rapidly consumed by the methanogenic bacterial consortium in the water column and/or is deflected laterally by bottom currents^48^. Therefore, we suggest that the anomalously negative *δ*^13^C values in the foraminiferal shells of core GeoB6201-5 were partially affected by post-depositional authigenic carbonate overgrowth. Elemental analyses of *C*. *wuellerstorfi* from core GeoB6201-5 reveals an elevated content of magnesium (Mg) and sulfur (S) between 140 and 235 cm core depth (supplementary Table S3), which is supported by bulk sediment mineralogy that shows the presence of high-Mg calcite (HMC) at the same interval (Fig. 2). Elevated Mg and S as well as the presence of HMC closely matches the interval of depleted-*δ*^13^C in foraminifera (Fig. 2 and Table S3), confirming that authigenic precipitation affected the original *δ*^13^C of foraminiferal shells. However, not all samples containing elevated Mg, S and HMC were associated with depleted *δ*^13^C. For example, shells analyzed at 195 cm core depth show *δ*^13^C values of −1.39‰ and an absence of Mg and S (supplementary Table S3), suggesting that the anomalously negative *δ*^13^C value represents the original test composition. On the other hand, mineralogical analyses indicated HMC in sediments at 180 cm core depth (Fig. 2 and supplementary Fig. S5), while foraminiferal shells show positive *δ*^13^C values together with high Mg (1.36%) and S (6.14%) (Supplementary Table S3). Our results suggest that AOM-derived authigenic carbonate precipitation may have added negative *δ*^13^C to some of the original foraminiferal shells, but this process was not responsible for all depleted *δ*^13^C values. Thus, the depleted-*δ*^13^C signals in core GeoB6201-5 evidence methane outgassing from the SBM, that occurred during the last glacial.

The AOM-depth in sediments at seep sites is mostly a function of fluid flow intensity; high flux displaces the AOM towards the seafloor while the AOM occurs deeper within sediments affected by low flux^49^. Authigenic carbonates containing aragonite (*i*.*e*. nodules, concretions and crusts) precipitate preferentially when the AOM occurs near the sediment-water interface due to a high methane flux^9,49,50^. No evidence of aragonite was observed in core GeoB6201-5, but the absence of aragonite in sediments can also be associated with post-depositional dissolution. Modeling experiments reveal that dissolution of aragonite in sediments starts just after the decrease in the methane flux and the halt in AOM, and ca. 2000 years would cause complete aragonite dissolution^51^. In addition, AOM-derived authigenic carbonates in methane seepage sites are generally enriched in *δ*^18^O^9,49,52,53^, which is not observed in our results (Fig. S6). Adjusting the *δ*^18^O values of *Uvigerina* spp. to the *Cibicides* spp. scale by subtracting 0.47‰^54^, glacial *δ*^18^O suggest that both taxa recorded the bottom water conditions (supplementary Fig. S6). This interpretation is supported by the similarity of the post-glacial *Cibicides* spp. *δ*^18^O curve of seep core GeoB6201-5 and non-seep core 14GGC^30^, collected at a similar water depth (Fig. S6a). Interestingly, an anomalous offset (of 0.2–2.72‰) is observed after the 17.5 cal ka BP (Fig. S6b). The origin of this offset is unclear. Yet, no depleted *δ*^13^C values is observed at this interval. It is unlikely that appreciable thermal gradient differences existed within the top few cm, and it is also unlikely that diagenetic processes can account for the isotopic offset of taxa that experienced the same postdepositional environment. It seems more likely that the decoupling of *δ*^18^O signals between the epifaunal *Cibicides* species and the infaunal *Uvigerina* species results from different ambient water conditions where they lived (bottom water compared with pore water). We therefore argue that such changes in the isotopic composition of benthic foraminifera reflect bottom water conditions at the time the shells were calcified. Further analyses of benthic foraminifera assemblages from core GeoB6201-5 are needed to confirm our interpretation of changes in seafloor conditions at the pockmark during the last glacial cycle. The observed depleted *δ*^13^C values in core GeoB6201-5 indicate that the AOM occurred close and/or at the seafloor, suggesting methane seepage from the SBM during the glacial.

Core GeoB6201-5 was collected from a pockmark depression on the SBM (26°S), where multi-channel seismic reflection profiles reveal shallow subsurface overpressured gas deposits accumulated in stratigraphic traps and gaps between pockmark arrays sensitive to hydrodynamic processes on the seafloor^5^. These deposits are connected to paleo-pockmarks through deep-seated complex subsurface faults, fractures and channels, highlighting a genetic relationship between salt diapirism and pockmarks^5^. During the last glacial (40–20 cal ka BP), global relative sea level was ca. 60–120 m lower than modern sea level^55^ (Fig. 3A). Hydrostatic depressurization over the sediment column probably contributed to expansion of the gas and the subsequent release of methane to the ocean. In addition to decreased hydrostatic pressure, the lower sea level displaced the upper water column structure downslope, bringing the core of the Brazil Current (BC) close to the core site location^56^. The BC increased local bottom temperatures, since surface and thermocline waters from the BC warmed up to 4 °C between 45 and 20 cal ka BP (Fig. 3B–C)^57,58^. Increased temperatures amplified the effect of decreased pressure on the shallow gas in subsurface sediments. The shift of the upper water column structure in relation to our core site is evident when the benthic *δ*^18^O record from core GeoB6201-5 (475 m water depth) is compared to the benthic *δ*^18^O record from adjacent core GeoB2107-3 located deeper in the water column (1084 m water depth) (Fig. S3e). The large glacial isotopic differences between benthic foraminifera from both cores (*δ*^18^O) suggests that core GeoB6201-5 was bathed by a warmer water mass while the core located deeper in the water column (GeoB2107-3) was exposed to a cooler water mass (Fig. 3E). In contrast, lower *δ*^18^O values occurring from the mid-deglaciation onwards suggest that both cores were bathed by a water mass with a similar *δ*^18^O signature. The downslope shift of the BC may also have intensified the bottom current velocity at site GeoB6201-5, which may have acted as a hydraulic pump^8,59^, facilitating the exhumation of gas methane from subsurface sediments to the ocean. The same mechanism was evoked to explain the pockmark formation in the Strait of Gibraltar (Mediterranean Sea) during the low sea level stand of the Last Glacial Maximum LGM)^8^.

After the LGM, the absence/reduced seepage of methane is evidenced by the more positive and less variable *δ*^13^C signals from core GeoB6201-5, with *δ*^13^C values becoming similar to those of modern foraminiferal calcite from non-seep environments (Figs 2 and 3B) (e.g. *Curry and Oppo*^39^ and *Chiessi et al*.^38^). The rising sea level increased the hydrostatic pressure on the seafloor and shifted the BC upslope decreasing bottom temperatures and current strength, thus reducing methane outgassing from the SBM (Fig. 3). The high sea level during the Holocene together with a decreased bottom current strength at our core site may have favored the deposition of pelagic fine-grained sediments, consequently forming a sealing layer that blocked the upward migration and allowed gas accumulation underneath the sediment, forming shallow subsurface deposits^5^. It is noteworthy that gas could also have migrated laterally within the sediments, not being recorded in our *δ*^13^C after the LGM, feeding nearby pockmarks as evidenced by current presence of chemosynthetic communities in this region^24^. This suggests that sea level changes and associated oceanographic processes played a key role on the stability of gas deposits in the SBM since ca. 40 cal ka BP.

Previously published evidence of past methane release from the Brazilian margin is limited to depleted *δ*^13^C values (−16 to −30‰) from authigenic carbonate nodules^26,27^. Some of these nodules from the southeastern Brazilian margin were U/Th-dated to 130–140 cal ka BP^27^, which are contemporaneous with an accumulation of warm water in the upper western South Atlantic Ocean that preceded Termination II^57,60^, as well as with a decrease in sea level^55^. Thus, available data are consistent with the notion that methane release from shallow subsurface gas deposits in the SBM occurred repeatedly over time, responding to glacial−interglacial changes in sea level and associated changes in the structure of the upper water column. The combination of lower sea level, warmer bottom waters and vigorous bottom current induced the destabilization of shallow subsurface gas deposits during the two recent glacial periods, while the higher sea level, relative cooling of bottom waters and reduced bottom current stabilized these gas deposits during the modern and last interglacials in the western South Atlantic Ocean.

In summary, we present the first foraminiferal *δ*^13^C records demonstrating methane outgassing in the SBM during the last glacial period. Our results show that shallow subsurface gas deposits in shallow water depths (ca. 500 m water depth) on the SBM are sensitive to changes in hydrostatic pressure, bottom water temperature and bottom current velocity over glacial-interglacial cycles. Additional research is needed to better understand the spatial-temporal paleoceanographic processes controlling methane gas dynamics and stability in the Brazilian margin. This is particularly important in the SBM where a huge hydrate deposit covering an area around 45.000 km^2^ with water depths ranging from 500 to 3500 m, the so-called Rio Grande Cone^23^, may have also been sensitive to the changes postulated in this study.

## Material and Methods

### Marine sediment cores

We examined core GeoB6201-5 (26°40′S, 46°26′W, 475 m water depth, 235 cm long)^28^, taken from the center of a funnel-shaped depression (600 m diameter and 70 m high) and core GeoB2107-3 (27°17′S, 46°45′W, 1048 m water depth, 780 cm long)^61^ collected outside of the pockmark, both from the SBM (Fig. 1). Core GeoB6201-5 is mainly composed of gray to very dark gray clay bearing nannofossil ooze, weakly to moderately bioturbated sediments with shell fragments and macrofossils^28^. Core GeoB 2107 (27°10,6′ S, 46°27,1′ W, 1048 m water depth), raised outside of the pockmark, consists of homogeneous dark grey nannofossil-bearing silt with two small sandy silt layers^61^.

### Stable isotopic analyses

Between 10 and 20 shells of planktonic foraminifera species *G.* *ruber* (white; sensu stricto) and *G*. *sacculifer* (with sac-like chamber) and 3–8 shells of benthic foraminifera *Cibicides* spp. (involving species *C*. *wuellerstorfi, C*. *lobatus and C*. *pachyderma*) and *Uvigerina* spp. (*U*. *peregrina* plexus) from core GeoB6201–5 as well as 10 and 20 shells of *G.* *ruber* and 3–8 shells of *Cibicides* spp. from core GeoB2107-3 (from the size fraction ≥ 250 μm) were selected for *δ*^13^C and *δ*^18^O analyses. Foraminiferal samples were analyzed on a Finnigan MAT 252 mass spectrometer equipped with an automatic carbonate preparation device at the MARUM/University of Bremen. Isotopic results were calibrated relative to the Vienna Peedee belemnite (VPDB) using the NBS19 standard. The standard deviation of the laboratory standard was lower than 0.07‰ and 0.05‰ for δ^18^O and δ^13^C, respectively, for the measuring period.

### Chronology of the sediment cores

The chronology of sediment core GeoB6201-5 is based on six AMS ^14^C measurements (supplementary Table S1), regional planktonic foraminifera biostratigraphy and oxygen isotope stratigraphy (supplementary Figs. S2, S3 and S4). The age model of GeoB2107-3 was published in Heil^36^ and Gu^37^.

### AMS ^14^C ages

The AMS ^14^C measurements of the planktonic foraminifera *G.* *ruber* from GeoB6201-5 were performed at the Poznan Radiocarbon Laboratory (supplementary Table. S1). To avoid possible contamination from an old carbon source, the samples selected for ^14^C analyses were chosen among the depths not affected by anomalous negative *δ*^13^C values, except for the basal age (235 cm). The age model and associated uncertainties were calculated using the R script BACON version 2.2^31^ and the IntCal13 calibration curve^32^ with a reservoir correction of 400 ± 100 years (supplementary Fig. S2). Beside the default parameters of the software, we used the following settings: mem.mean = 0.7, acc.shape = 0.8. and t.a = 8/t.b = 9. Sedimentation rates for core GeoB6201-5 are shown in supplementary Fig. S3.

### Verifying the ^14^C-based chronostratigraphy of core GeoB6201-5 with foraminiferal oxygen stable isotopes

We verified the ^14^C-based age model of GeoB6201-5 by comparing our benthic *Cibicides* spp. *δ*^18^O record with the *Cibicides* spp. *δ*^18^O record from adjacent well-dated core GeoB2107-3^36,37^ and the intermediate South Atlantic benthic *δ*^18^O stack LS16^33^ (supplementary Fig. S3a). This approach supports the ^14^C-based age model indicating that core GeoB6201-5 recorded the last 40 cal ka BP, corresponding to Marine Isotope Stages 1–3 (supplementary Fig. S4a).

### Verifying the ^14^C-based chronostratigraphy of GeoB6201–5 with planktonic foraminifera biostratigraphy

The presence/absence of planktonic foraminifera species and subspecies of *Globorotalia menardii*, the so-called “menardiform plexus”, in marine cores has been widely applied as an indicator of paleoclimatic fluctuations of the late Quaternary, and the menardiform plexus is the main group of planktonic foraminiferal species used in the biostratigraphic zonation of this interval^34,35^. Ericson and Wollin^34^ divided the Late Quaternary into 10 biozones (Q to Z) in accordance with the presence/absence of the menardiform plexus. The menardiform plexus was found to occur in the interglacial intervals (biozones R, T, V, and X), whereas its absence characterized the glacial intervals (biozones Q, S, U, W, and Y) of the late Pleistocene. As the Holocene is the latest interglacial, it was named zone Z (Fig. 1). In core GeoB6201-5, the absence of the menardiform plexus between 235 and 55 cm core depth (ca. 40–10 cal ka BP) is indicative of the glacial biozone Y of Ericson and Wollin^34^, while its presence after 55 cm core depth (ca. 10 cal ka BP) indicates the post-glacial biozone Z (Holocene) (supplementary Fig. S4b). In addition, the elevated abundance of *G*. *inflata*, a cold-transitional water species in the southwestern Atlantic Ocean^62^ being regionally abundant during glacial biozone Y and virtually absent during the Holocene biozone Z^35^, further supports the ^14^C-based age model of core GeoB6201-5 (supplementary Fig. S4b).

### Non-seep adjacent cores

We used a published record from adjacent core 14GGC (26°68’S, 46°50’W, 441 m water depth, 200 cm long) as a control core, in addition to GeoB2107-3. The *Cibicidoides wuellerstorfi δ*^13^C records and age model from core 14GGC were presented in Lund *et al*.^30^.

### Mineralogical analyses

The bulk mineralogy of 13 samples of core GeoB6201-5 was determined by X-ray diffraction (XRD) (Bruker D8 Advance using Cu Kα radiation) at the Physics Institute/Fluminense Federal University. The samples were powdered before analyses and the diffractograms were produced with 2θ from 3° to 70°, with an increment of 0.02° and 0.1 s of reading time. Minerals were identified by comparison of the experimental pattern with tables from Brindley and Brown^63^.

### Elemental composition of benthic foraminifera

Scanning electron microscope energy dispersive X-ray (SEM-EDX) analyses of 19 individual specimens of *Cibicidoides wuellerstorfi* (>150 μm) from 13 sediment samples of core GeoB6201-5 were carried out in the Institute of Chemistry/Fluminense Federal University. The analyzed specimens were cleaned by sonication for 20 s in methanol and subsequently in distilled water to remove the methanol. The samples were dried for 24 hours at 40 °C, put on carbon adhesive tape in a circular 25 mm diameter stub, and analyzed on a SEM Hitachi Analytical Table Top Microscope/benchtop SEM TM3000 equipped with a EDX detector by Bruker Nano GmbH. The EDX point analyses were obtained from the penultimate chamber of each specimen. For optimal results, a minimum of one minute for each target was selected after rarefaction optimization of results from 6, 4, 2 and 1 minute minimum.

## Electronic supplementary material

Supplementary information
