# Supplementary material for: Methane release from the southern Brazilian margin during the last glacial
Source: Sci Rep. 2018 Apr 13;8:5948. doi: 10.1038/s41598-018-24420-0 (PMC5899165; doi:10.1038/s41598-018-24420-0)
Supplement: Supplementary file 1 — Supplementary information [file 41598_2018_24420_MOESM1_ESM.doc]

**Methane release from the southern Brazilian margin during the last glacial**

**Portilho-Ramos, R.C.^1,2,3^, Cruz, A.P.S.^2^, Barbosa, C.F.^2^, Rathburn, A.E.^4^, Mulitza, S.^1^, Venancio, I.M.^5^, Schwenk, T.^1^, Rühlemann, C.^6^, Vidal, L.^7^, Chiessi, C.M.^8^, Silveira, C.S.^2^.**

**1. Supplementary information**

**1.1. Supplementary Regional Setting**

The southeastern Brazilian margin (SBM) is under the influence of the western boundary Brazil Current (BC), which transport tropical waters southward along the Brazilian coast (Fig. 1a). The BC originates around 15°S after the South Equatorial Current (SEC) splits into the southward flowing Brazil Current (BC), and the northward flowing NBC (Fig. 1a)^1,2^. The BC is relatively weak and receives ca. 4Sv (1Sv = 10^6^ m^3^s^-1^) in comparison to the NBC that receives ca. 12Sv from the SEC^3^. The volume transported by the BC does not change significantly between 10°–20°S^3,4^. At 20°S, however, the BC receives contributions from South Atlantic Central Water (SACW) and becomes deeper, reaching 750 m in thickness at 28°S^5^.

Three water mass can be identified in the upper 1100 m in southwestern Atlantic Ocean, the Tropical Waters (TW), the SACW and the Antarctic Intermediated Water (AAIW) (Fig. S1). The TW is warm (≥ 20^o^C) and salty (≥ 36.4psu) water transported southward by the BC^5^. Underneath the TW, the SACW is located mainly between 200 and 500 m water depth, characterized by temperatures of 5–20^o^C and salinity of 34.6 – 36psu^6^ (Fig. S1a-b). It is formed at the southern boundary of the south Atlantic subtropical gyre (Subtropical Confluence – STC), particularly at the Brazil–Malvinas Confluence (BMC) around 38°S – 45°S^7^, but also receives a contribution of thermocline waters from the Indian Ocean through the Agulhas leakage corridor^7,8^. South of 20^o^S, SACW is transported southward by BC^5^. The AAIW is characterized by temperatures (≤ 6^o^C) and salinity (≤ 34.6psu)^6^, and flow northward underneath the SACW (Fig. S1a-b).

**2. Stable carbon isotopes (δ^13^C) from foraminiferal shells as evidenced for modern and past methane cold- seepage**

Stable carbon isotopes (δ^13^C) from foraminiferal carbonate have been widely used as a proxy for interpreting methane releasing through destabilization of hydrates deposits in geological records^9–12^. Calcareous tests (shells) of benthic foraminifera living in cold-seep habitats have, on average, more negative δ^13^C values compared to the same species living in non-seep environments^13–18^ (Table S2). No benthic foraminifera species are endemic to cold seeps^13,16^, and recent genetic work on one seep species supports the conclusion from morphologic taxonomy that seep species are cosmopolitan and not different from non-seep congeners^19^. Although isotopic signatures of foraminifera are used extensively in paleoceanographic applications and are known to be influenced by ambient conditions, the processes behind the incorporation of negative δ^13^C signals by seep foraminifera are still not well constrained^12,14,20–22^. The mechanism behind the incorporation of δ^13^C-deplection by benthic foraminifera has been attributed to consumption of organic matter with negative δ^13^C^14,15^ and postdepositional overgrowth of authigenic calcite^23–25^. Recent laboratory culture experiments using pressure chambers to expose deep-water species to labeled methane showed that these species can live in a methane-laden environment, and that the carbon isotopic composition of calcareous benthic foraminiferal tests are influenced by δ^13^C values of methane^18^. As a result of the relationship between δ^13^C values of carbonate from living foraminifera and exposure to seafloor methane, anomalously negative δ^13^C excursions in fossil foraminiferal carbonate from sedimentary records are used to assess the geologic history of methane seepage^9–12,26^.

Negative δ^13^C values in foraminifera shells are likely to be affected by postdepositional contamination by authigenic carbonate with light δ^13^C_DIC_ derived by Anaerobic Oxidation of Methane (AOM) in bottom-influenced waters^23–25^. Authigenic crusts (diagenetic process) derived from AOM can account for substantial (3% to 20%) depletion in the δ^13^C values of foraminiferal carbonate cold seep environments^20,24,25,27,28^. Authigenic carbonate precipitation is stimulated by the increased alkalinity of the water generated by AOM in surface sediments (sediment-ocean interface) and/or inside the sediment column at/or under a thin layer Sulfate-Methane Interface^29^. In general, high rates of AOM occur in methane-rich sediments near the sediment-water interface^29^. The presence of diagenetic calcite might not be visible in scanning electron microscope examination and after ultrasonic cleaning process as frequently reported in literature^14,20,23,25,27^.

**2.1. Gas methane deposits and cold seeps along the Brazilian continental margin**

Hydrates reservoir and free-gas underneath the sediment has been reported offshore along the brazilian continental margin^30–34^ which has been shown to be composed basically by methane gas (>99.78%)^35^. The largest hydrate reserve is located in southern Brazil, the Rio Grande Cone in Pelotas Basin, covering an area of 45,000 km^2^ with water depths ranging from 500 to 3000 m^30,33,35^. The recent discovery of chemosynthetic community (*e.i.* anaerobic methanotrophic archaeal groups and bivalve *Acharax* sp.), high methane concentrations and pockmarks associated to salt diapirs on the seafloor are indicative of active cold methane seeps on the southern Brazilian margin^34^. Several circular depressions of collapsed sediments along the brazilian continental shelf and slope are suggestive of past cold-methane seeps activity[*Schulz et al*., 2001; *Schattner et a*l., 2016]. These structures show ca. 600−1000m of diameters and 40−70m depth and are distributed parallel to shelf break between 300−1500m water depth^35–37^. Recent multi-channel seismic reflection profiles revels co-occurrence of hydrocarbon source, salt diapirs fields and pockmark structures, indicating the genetic relationship between pockmarks and diapirism in Brazilian margin^37^. These data show a complex subsurface structures of faults, fractures and channels that connect these shallow free gas deposits to deep-seated salt diapirs and paleo-pockmarks (deep sedimentary unit of organic-rich sediments from Late Miocene), suggesting deep-source of gas may have formed and fed the local pockmarks through geological time^37^. In accordance to Mahiquens et al^37^, salt diapirs and associated faults work as conduits of gas/fluids and facilitating its upward migration through faults from deep-seated source to shallow subsurface reservoir where it is retained in stratigraphic traps and gaps between pockmark arrays in shallow depths subsurface; therefore increasing the pore pressure. These deposits are highly sensitive to hydrodynamic process on the sea floor (*e.i.* effective hydrostatic pressure stress and changes in speed of the bottom currents)^37^.

**3. Supplementary References**

1. Combes, V. & Matano, R. P. A two-way nested simulation of the oceanic circulation in the Southwestern Atlantic. *J. Geophys. Res. Ocean.* **119,** 731–756 (2014).

2. Peterson, R. G. *et al.* Upper-level circulation in the South Atlantic Ocean. *Prog. Oceanogr.* **26,** 1–73 (1991).

3. Stramma, L., Ikeda, Y. & Peterson, R. G. Geostrophic transport in the Brazil current region north of 20°S. *Deep Sea Res. Part A. Oceanogr. Res. Pap.* **37,** 1875–1886 (1990).

4. Peterson, R. G. & Stramma, L. Upper-level circulation in the South Atlantic Ocean. *Prog. Oceanogr.* **26,** 1–73 (1991).

5. Silveira, I. C. A. da, Schmidt, A. C. K., Campos, E. J. D., Godoi, S. S. de & Ikeda, Y. A corrente do Brasil ao largo da costa leste brasileira. *Brazilian J. Oceanogr.* **48,** (2000).

6. Stramma, L. & Schott, F. The mean flow field of the tropical Atlantic Ocean. *Deep Sea Res. Part II Top. Stud. Oceanogr.* **46,** 279–303 (1999).

7. Stramma, L. & England, M. On the water masses and mean circulation of the South Atlantic Ocean. *J. Geophys. Res.* **104,** 20863–20883 (1999).

8. Biastoch, A., Böning, C. W., Schwarzkopf, F. U. & Lutjeharms, J. R. E. Increase in Agulhas leakage due to poleward shift of Southern Hemisphere westerlies. *Nature* **462,** 495–8 (2009).

9. Kennett, J. P. Carbon Isotopic Evidence for Methane Hydrate Instability During Quaternary Interstadials. *Science (80-. ).* **288,** 128–133 (2000).

10. Hill, T. M., Paull, C. K. & Critser, R. B. Glacial and deglacial seafloor methane emissions from pockmarks on the northern flank of the Storegga Slide complex. *Geo-Marine Lett.* **32,** 73–84 (2012).

11. Panieri, G. *et al.* Late Holocene foraminifera of blake ridge diapir: Assemblage variation and stable-isotope record in gas-hydrate bearing sediments. *Mar. Geol.* **353,** 99–107 (2014).

12. Millo, C., Sarnthein, M., Erlenkeuser, H. & Frederichs, T. Methane-driven late Pleistocene d13C minima and overflow reversals in the southwestern Greenland Sea. *Geology* **33,** 873–876 (2005).

13. Rathburn, A. E. *et al.* Relationships between the distribution and stable isotopic composition of living benthic foraminifera and cold methane seep biogeochemistry in Monterey Bay, California. *Geochemistry, Geophys. Geosystems* **4,** (2003).

14. Hill, T. M., Kennett, J. P. & Valentine, D. L. Isotopic evidence for the incorporation of methane-derived carbon into foraminifera from modern methane seeps, Hydrate Ridge, Northeast Pacific. *Geochim. Cosmochim. Acta* **68,** 4619–4627 (2004).

15. Mackensen, A., Wollenburg, J. & Licari, L. Low D 13 C in tests of live epibenthic and endobenthic foraminifera at a site of active methane seepage. **21,** 1–12 (2006).

16. Bernhard, J. M., Martin, J. B. & Rathburn, A. E. Combined carbonate carbon isotopic and cellular ultrastructural studies of individual benthic foraminifera: 2. Toward an understanding of apparent disequilibrium in hydrocarbon seeps. *Paleoceanography* **25,** 1–12 (2010).

17. Gieskes, J. *et al.* Cold seeps in Monterey Bay, California: Geochemistry of pore waters and relationship to benthic foraminiferal calcite. *Appl. Geochemistry* **26,** 738–746 (2011).

18. Wollenburg, J. E., Raitzsch, M. & Tiedemann, R. Novel high-pressure culture experiments on deep-sea benthic foraminifera - Evidence for methane seepage-related δ13C of Cibicides wuellerstorfi. *Mar. Micropaleontol.* **117,** 47–64 (2015).

19. Burkett, A. M. *et al.* Phylogenetic placement of Cibicidoides wuellerstorfi (Schwager, 1866) from methane seeps and non-seep habitats on the Pacific margin. *Geobiology* **13,** 44–52 (2015).

20. Torres, M. E. Is methane venting at the seafloor recorded by δ 13 C of benthic foraminifera shells? *Paleoceanography* **18,** 1062 (2003).

21. Herguera, J. C., Paull, C. K., Perez, E., Ussler, W. & Peltzer, E. Limits to the sensitivity of living benthic foraminifera to pore water carbon isotope anomalies in methane vent environments. *Paleoceanography* **29,** 273–289 (2014).

22. Eberwein, a. & Mackensen, a. Regional primary productivity differences off Morocco (NW-Africa) recorded by modern benthic foraminifera and their stable carbon isotopic composition. *Deep. Res. Part I Oceanogr. Res. Pap.* **53,** 1379–1405 (2006).

23. Torres, M. E., Martin, R. a., Klinkhammer, G. P. & Nesbitt, E. a. Post depositional alteration of foraminiferal shells in cold seep settings: New insights from flow-through time-resolved analyses of biogenic and inorganic seep carbonates. *Earth Planet. Sci. Lett.* **299,** 10–22 (2010).

24. Panieri, G. *et al.* Diagenetic Mg-calcite overgrowths on foraminiferal tests in the vicinity of methane seeps. *Earth Planet. Sci. Lett.* **458,** 203–212 (2017).

25. Millo, C., Sarnthein, M., Erlenkeuser, H., Grootes, P. M. & Andersen, N. Methane-induced early diagenesis of foraminiferal tests in the southwestern Greenland Sea. *Mar. Micropaleontol.* **58,** 1–12 (2005).

26. Smith, L. M., Sachs, J. P., Jennings, A. E., Anderson, D. M. & DeVernal, A. Light δ 13 C events during deglaciation of the East Greenland Continental Shelf attributed to methane release from gas hydrates. *Geophys. Res. Lett.* **28,** 2217–2220 (2001).

27. Martin, R. a., Nesbitt, E. a. & Campbell, K. a. The effects of anaerobic methane oxidation on benthic foraminiferal assemblages and stable isotopes on the Hikurangi Margin of eastern New Zealand. *Mar. Geol.* **272,** 270–284 (2010).

28. Cook, M. S., Keigwin, L. D., Birgel, D. & Hinrichs, K.-U. Repeated pulses of vertical methane flux recorded in glacial sediments from the southeast Bering Sea. *Paleoceanography* **26,** n/a-n/a (2011).

29. Ussler, W. & Paull, C. K. Rates of anaerobic oxidation of methane and authigenic carbonate mineralization in methane-rich deep-sea sediments inferred from models and geochemical profiles. *Earth Planet. Sci. Lett.* **266,** 271–287 (2008).

30. Fontana, R. L. & Mussumeci, a. Hydrates offshore Brazil. *Ann. N. Y. Acad. Sci.* **715,** 106–113 (1994).

31. Maslin, M., Mikkelsen, N., Vilela, C. & Haq, B. Sea-level –and gas-hydrate–controlled catastrophic sediment failures of the Amazon Fan. *Geology* **26,** 1107 (1998).

32. Kowsmann, R. O. & De Carvalho, M. D. Erosional event causing gas-venting on the upper continental slope, Campos Basin, Brazil. *Cont. Shelf Res.* **22,** 2345–2354 (2002).

33. Oliveira, S., Vilhena, O. & da Costa, E. Time–frequency spectral signature of Pelotas Basin deep water gas hydrates system. *Mar. Geophys. Res.* **31,** 89–97 (2010).

34. Giongo, A. *et al.* Discovery of a Chemosynthesis-based Community in the Western South Atlantic Ocean. *Deep Sea Res. Part I Oceanogr. Res. Pap.* (2015). doi:10.1016/j.dsr.2015.10.010

35. Miller, D. J. *et al.* Natural gas hydrates in the Rio Grande Cone (Brazil): A new province in the western South Atlantic. *Mar. Pet. Geol.* **67,** 187–196 (2015).

36. Schattner, U., Lazar, M., Souza, L. A. P., ten Brink, U. & Mahiques, M. M. Pockmark asymmetry and seafloor currents in the Santos Basin offshore Brazil. *Geo-Marine Lett.* **36,** 457–464 (2016).

37. Mahiques, M. M., Schattner, U., Lazar, M., Sumida, P. Y. G. & Souza, L. A. P. De. An extensive pockmark field on the upper Atlantic margin of Southeast Brazil: spatial analysis and its relationship with salt diapirism. *Heliyon* **3,** (2017).

38. Locarnini, R. A. et al. World Ocean Atlas 2009. In: Levitus, S. (Ed.), temperature, NOAA Atlas NESDIS 68 vol. 1. U.S. Government Printing Office, Washington, D. C., 184 pp. (2010).

39. Antonov, J. I. et al. World Ocean Atlas 2009. In: Levitus, S. (Ed.), Salinity, NOAA Atlas NESDIS 69 vol. 2. U.S. Government Printing Office, Washington, D. C., 184 pp. (2010).

40. Schlitzer, R. Ocean Data View. http://odv.awi.de (2017).

41. Hendry, K. R. *et al.* Abrupt changes in high-latitude nutrient supply to the Atlantic during the last glacial cycle. *Geology* **40,** 123–126 (2012).

42. Lisiecki, L. E. & Stern, J. V. Regional and global benthic D18 O stacks for the last glacial cycle. *Paleoceanography* **31,** 1368–1394 (2016).

43. Ericson, D. B. & Wollin, G. Pleistocene Climates and Chronology in Deep-Sea Sediments. *Science (80-. ).* **162,** 1227–1234 (1968).

44. Blaauw, M. & Christeny, J. A. Flexible paleoclimate age-depth models using an autoregressive gamma process. *Bayesian Anal.* **6,** 457–474 (2011).

45. Marchitto, T. M. *et al.* Improved oxygen isotope temperature calibrations for cosmopolitan benthic foraminifera. *Geochim. Cosmochim. Acta* **130,** 1–11 (2014).

**Table S1**: Radiocarbon AMS dates (reservoir corrected using Bacon database) used in age model reconstruction.

| **Core** | **Sample code** | **Depth (cm)** | **Material** | **^14^C age (ka)** | **Error (ka)** | **Age (ka)** | **Error (ka)** |
| --- | --- | --- | --- | --- | --- | --- | --- |
| **GeoB6201-5** | Poz-32139 | 0 | *G. ruber* | 2.47 | ±0.030 | 2.56 | ±0.097 |
| **GeoB6201-5** | Poz-32140 | 55 | *G. ruber* | 9.69 | ±0.050 | 11.13 | ±0.082 |
| **GeoB6201-5** | Poz-32141 | 95 | *G. ruber* | 14.31 | ±0.080 | 16.82 | ±0.153 |
| **GeoB6201-5** | Poz-32142 | 145 | *G. ruber* | 18.86 | ±0.110 | 22.44 | ±0.116 |
| **GeoB6201-5** | Poz-32143 | 180 | *G. ruber* | 19.18 | ±0.110 | 22.78 | ±0.149 |
| **GeoB6201-5** | Poz-32144 | 235 | *G. ruber* | 39.00 | ±1.000 | 43.86 | ±0.886 |

| **Species** | **δ^13^C** | **Seeps location** | **Time-scale** | **References** |
| --- | --- | --- | --- | --- |
| ***Cibicides* spp*.*** | −3.20‰ − −0.31‰ | Off southeastern Brazil in SW-Atlantic | Last glacial (20kyr – 40kyr ) | This study |
| ***Cibicides mckannai*** | −2.28‰ − −0.41‰ | Santa Barbara Channel in NE−Pacific | Last glacial (21kyr – 35kyr) | Hill et al. (2003) |
| ***Cibicides lobatulus*** | −5.80‰ − 1.21‰ | Off southwestern Greenland Sea | Last glacial (40kyr – 90kyr) | Millo et al. (2005a) |
| ***Cibicides wuellestorfi*** | −3.30‰ − 2.10‰ | Monterrey Bay in NE-Pacific | Modern seep | Bernhard et al. (2010) |
| ***Uvigerina* spp*.*** | −4.60‰ − −0.12‰ | Off southeastern Brazil in SW-Atlantic | Last glacial (20kyr – 40kyr ) | This study |
| ***Uvigerina peregrina*** | −2.36‰ − −1.15‰ | Santa Barbara Channel in NE−Pacific | Last glacial (21kyr – 35kyr) | Hill et al. (2003) |
| ***Uvigerina peregrina*** | −4.20‰ − −0.80‰ | Blake Ridge diaper in NW-Atlantic | Late Holocene | Panieri et al. (2014) |
| ***Uvigerina peregrina*** | −2.20‰ − 0.90‰ | Monterrey Bay in NE-Pacific | Modern seep | Bernhard et al. (2010) |
| ***Uvigerina peregrina*** | −2.70‰ − −0.70‰ | Hikurangi Margin in New Zealand | Modern seep | Martin et al. (2010) |
| ***Uvigerina peregrina*** | −1.53‰ − −0.51‰ | Off Eel River mouth in northern California | Modern seep | Rathburn et al. (2000) |
| ***Uvigerina peregrina*** | −2.05‰ − −0.10‰ | Monterrey Bay in NE-Pacific | Modern seep | Rathburn et al. (2003) |

**Table S2:** Benthic foraminifera δ^13^C values from modern and fossil cold seep environments.

**Table S3:** Scanning Electron Microscope energy-dispersive X-ray (SEM-EDX) qualitative estimative of major trace element (dry weight %) content in benthic foraminifera species *Cibicides* *wuellerstofi* from core GeoB6201-5.

| **Depth (cm)** | **Species** | **δ^18^O (‰)** | **δ^13^C (‰)** | **C (%)** | **O (%)** | **S (%)** | **Ca (%)** | **K (%)** | **Al (%)** | **Ch (%)** | **Si (%)** | **S (%)** | **Mg (%)** |
| --- | --- | --- | --- | --- | --- | --- | --- | --- | --- | --- | --- | --- | --- |
| **20cm (I)** | *C. wuellerstofi* | 2,85 | 1,2 | 20,83 | 51,25 | 0,72 | 27,20 | - | - | - | - | - | - |
| **20cm (II)** | *C. wuellerstofi* | 2,85 | 1,2 | - | 56,29 | 1,07 | 41,94 | 0,70 | - | - | - | - | - |
| **20cm (III)** | *C. wuellerstofi* | 2,85 | 1,2 | - | 52,22 | 1,54 | 44,29 | - | 0,70 | 1,26 | - | - | - |
| **40cm (I)** | *C. wuellerstofi* | 1,55 | 2,08 | 18,18 | 52,11 | 0,70 | 29,00 | - | - | - | - | - | - |
| **40cm (II)** | *C. wuellerstofi* | 1,55 | 2,08 | 19,73 | 53,24 | - | 27,03 | - | - | - | - | - | - |
| **40cm (III)** | *C. wuellerstofi* | 1,55 | 2,08 |  | 62,60 | 0,96 | 32,21 | 2,31 |  | 1,93 | - | - | - |
| **60cm(I)** | *C. wuellerstofi* | 2,53 | 0,96 | 21,04 | 56,19 |  | 22,55 | - | - | - | 0,21 | - | - |
| **60cm(II)** | *C. wuellerstofi* | 2,53 | 0,96 | - | 68,50 | 0,37 | 29,81 | 0,28 | 0,34 | - | 0,70 | - | - |
| **80cm (I)** | *C. wuellerstofi* | 2,47 | 1,12 | 18,74 | 50,55 | - | 30,49 | - | - | - | 0,22 | - | - |
| **100cm (I)** | *C. wuellerstofi* | 3,02 | 0,74 | 17,24 | 43,15 | - | 39,33 | - | - | - | 0,28 | - | - |
| **120cm (I)** | *C. wuellerstofi* | 3,06 | 0,33 | - | 63,42 | 0,83 | 35,05 | 0,31 | - | - | 0,39 | - | - |
| **140cm (I)** | *C. wuellerstofi* | 2,6 | -0,76 | 19,92 | 28,42 |  | 50,99 |  | 0,34 |  | 0,33 | - | - |
| **140cm (II)** | *C. wuellerstofi* | 2,6 | -0,76 | - | 54,00 | 1,00 | 42,44 | 1,05 | - | 0,70 | 0,30 | 0,50 | - |
| **160cm (I)** | *C. wuellerstofi* | 2,6 | 0,23 | 22,14 | 31,40 | - | 45,07 | - | 0,40 | - | 0,47 | 0,27 | 0,25 |
| **160cm (II)** | *C. wuellerstofi* | 2,6 | 0,23 | 17,12 | 52,45 | - | 27,90 | - | 0,27 | - | 0,27 | - | 1,99 |
| **180cm (I)** | *C. wuellerstofi* | 3,01 | 0,19 | - | 57,75 | 0,46 | 32,65 | 0,41 | 0,77 | - | 0,46 | 6,14 | 1,36 |
| **195cm (I)** | *C. wuellerstofi* | 2,62 | -1,39 | 22,61 | 55,914 | 0,303 | 20,621 | - | 0,259 | - | 0,293 | - | - |
| **210cm (I)** | *C. wuellerstofi* | 2,5 | -2,59 | - | 62,58 | 0,88 | 32,99 | 0,44 | 0,46 | 0,32 | 0,79 | - | 1,56 |
| **220cm (I)** | *C. wuellerstofi* | 2,63 | -0,06 | - | 65,04 | - | 31,94 | 0,26 | 0,36 | - | - | - | 2,39 |
| **235cm (I)** | *C. wuellerstofi* | 2,64 | -1,67 | - | 63,77 | - | 28,69 | 0,67 | 2,16 | - | 3,20 | - | 1,01 |

**FIGURES CAPTION**

**
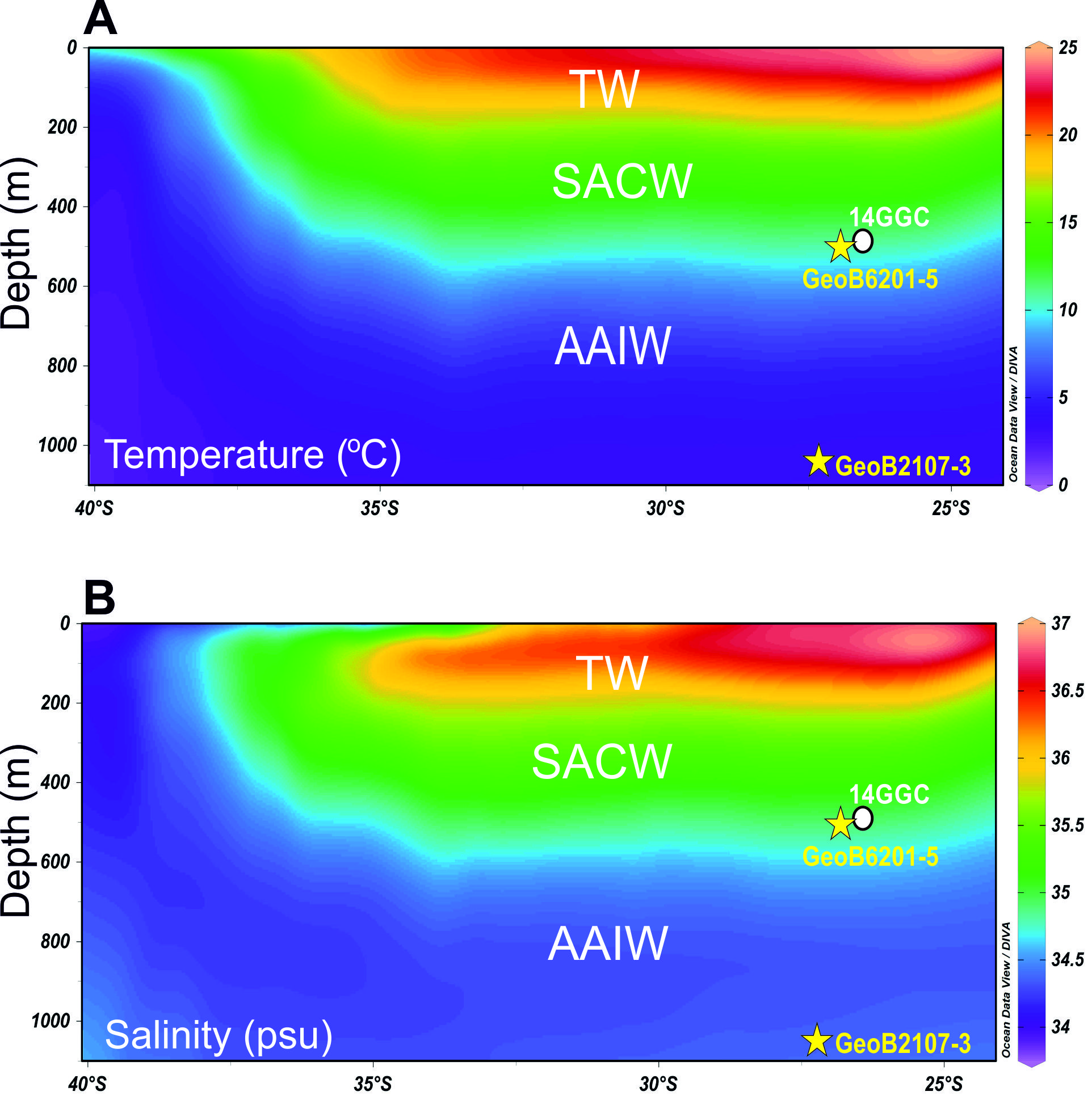
**

**Figure S1:** Main water masses in the southwestern Atlantic Ocean in the upper 1100m water depth. (**A**) Temperature^38^ and (**B**) Salinity^39^. Tropical Waters (TW), the South Atlantic Central Waters (SACW) and the Antarctic Intermediated Water (AAIW). Figure created using Ocean Data View software^40^ (ODV - version, 4.7.9., http://odv.awi.de, 2017).


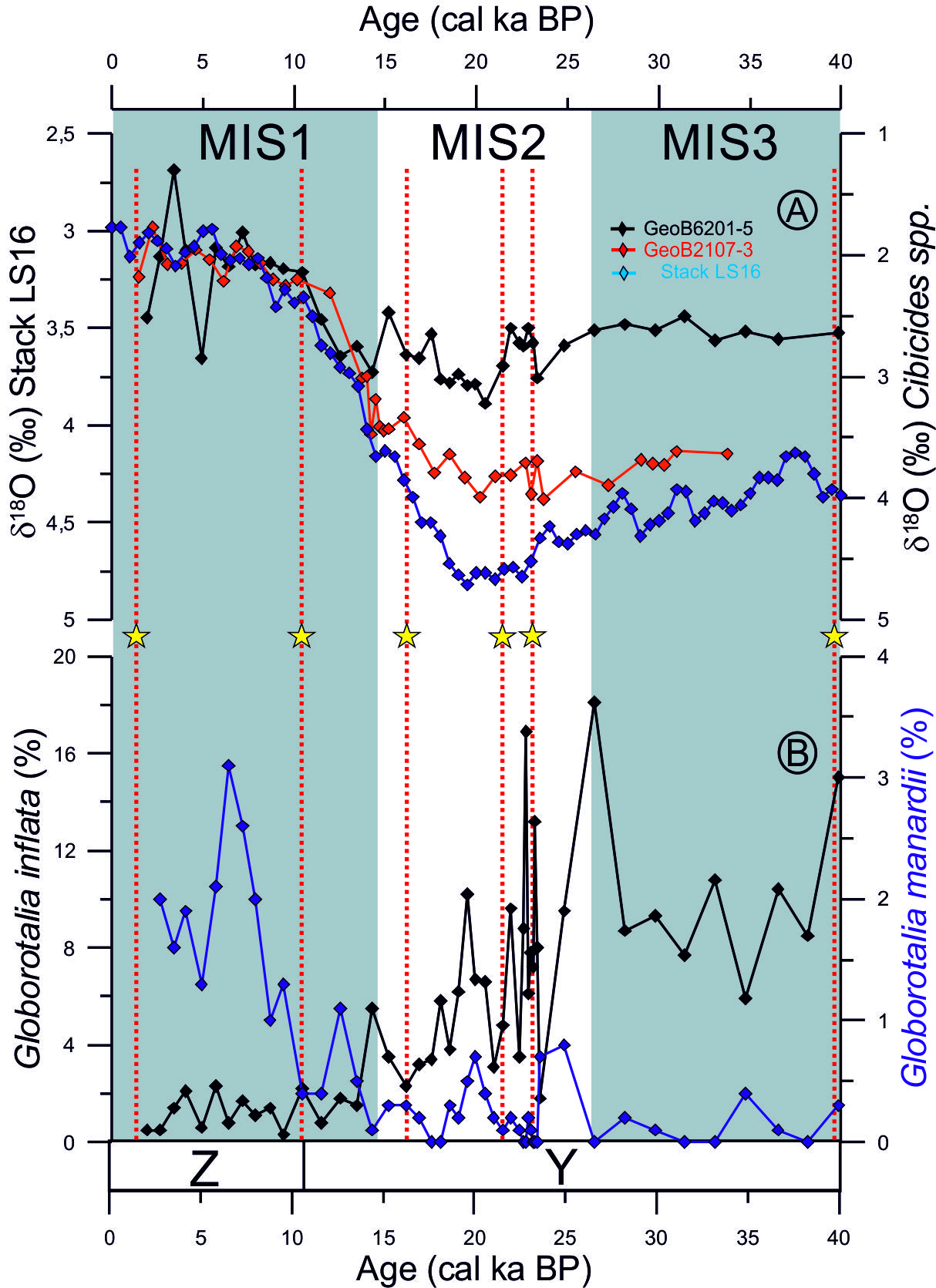


**Figure S2:** Age model of the core GeoB6205-1 based on the compilation of 6 AMS ^14^C dates (yellow stars), (**A**) Comparison of *Cibicides* spp. δ^18^O records of GeoB6205-1 and benthic δ^18^O records from adjacent core GeoB2107-3^41^ to intermediate South Atlantic benthic δ^18^O Stack LS16 [LS16;*Lisiecki and Ste*rn, 2016]. (B) Abundance of the main biostratigraphical planktonic foraminifera species *Globorotalia menardii* and *Globorotalia inflata* recorded in core GeoB6201-5. Marine Isotopic Stages 1 to 3 (MIS1–3) are shown at the top, while letters Z and Y in the bottom correspond to biostratigraphical biozones of Ericson and Wollin^43^.


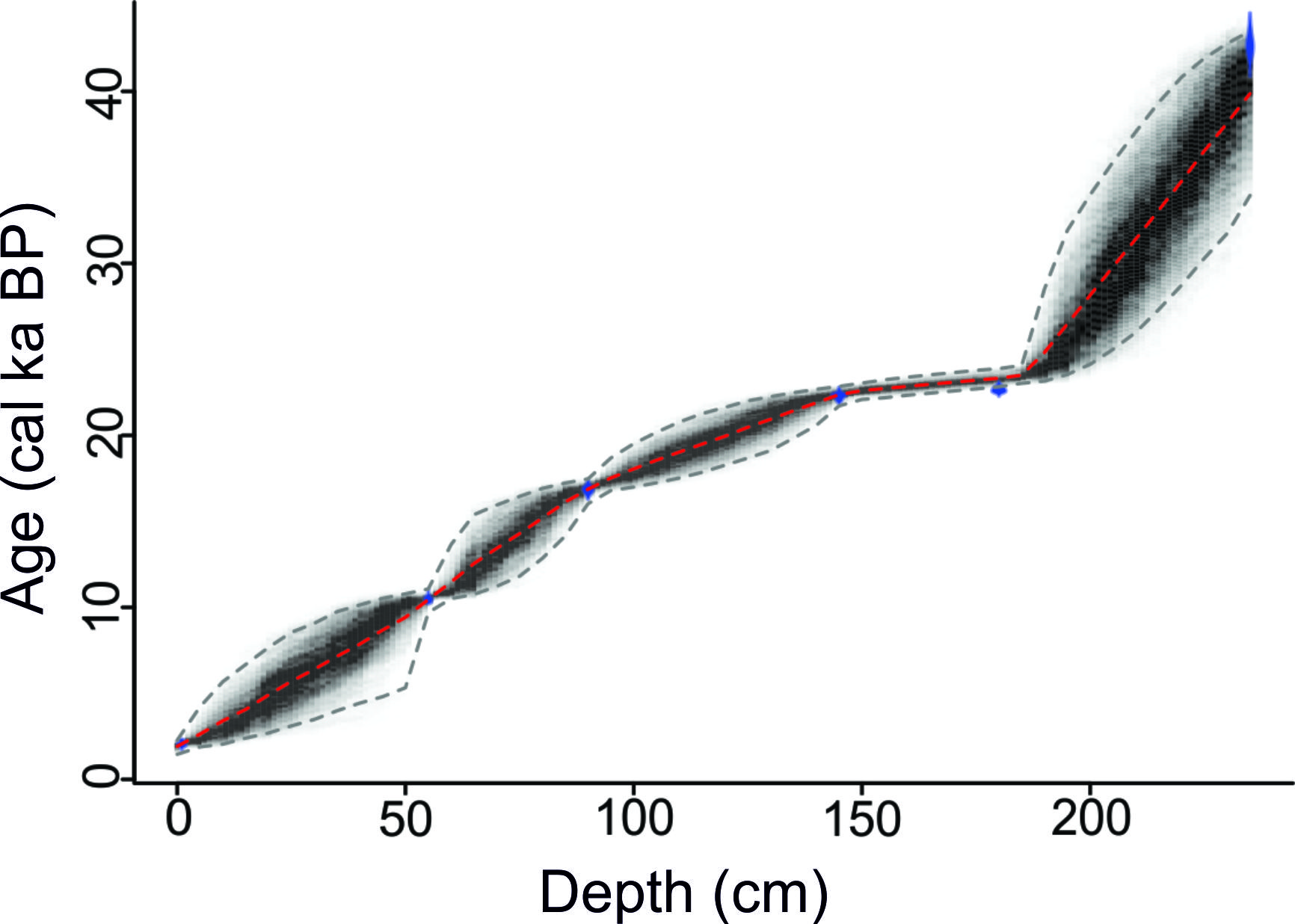


**Figure S3:** Age-depth model for core GeoB6201-5 based on Bacon v. 2.2^44^. The symbols represent the positions of the calibrated AMS ^14^C ages.


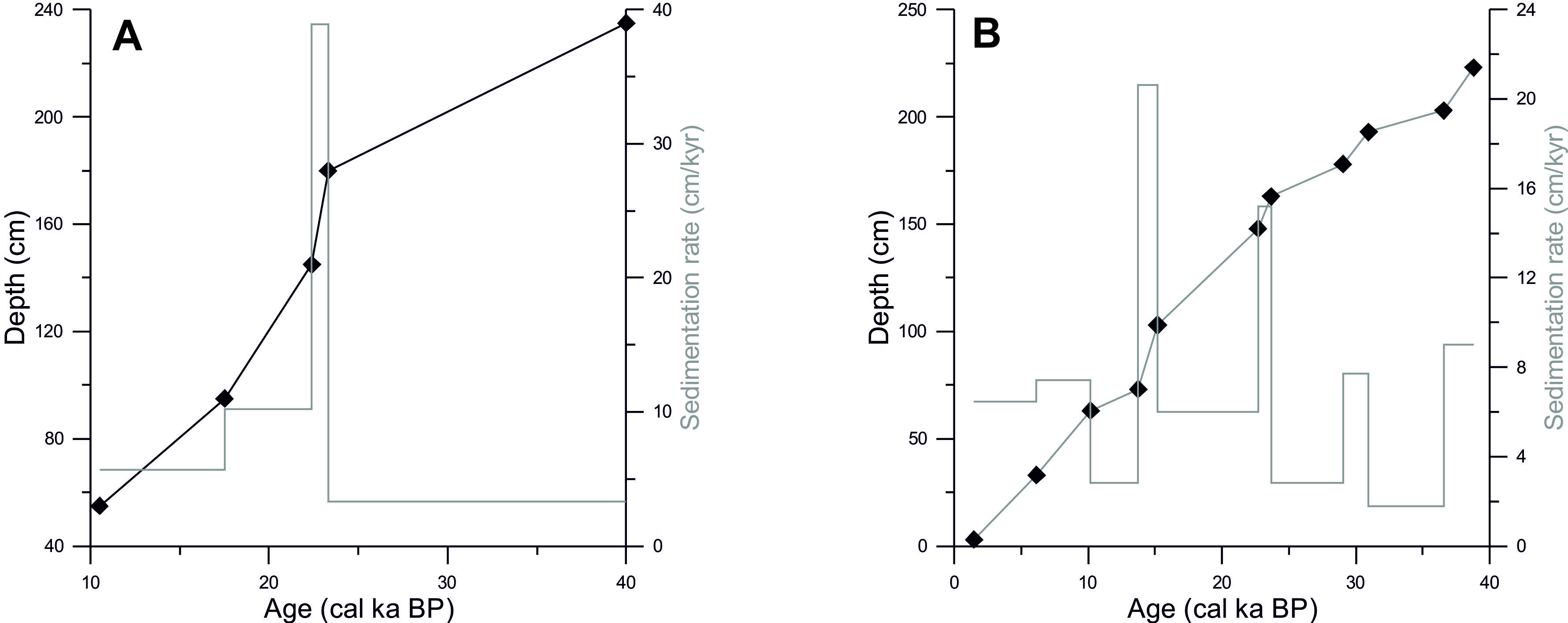


**Figure S4:** Age model (black line) and sedimentation rate (grey line) for (**A**) core GeoB6201-5 and (**B**) core GeoB2107-3. Black diamonds indicate calibrated AMS ^14^C ages.


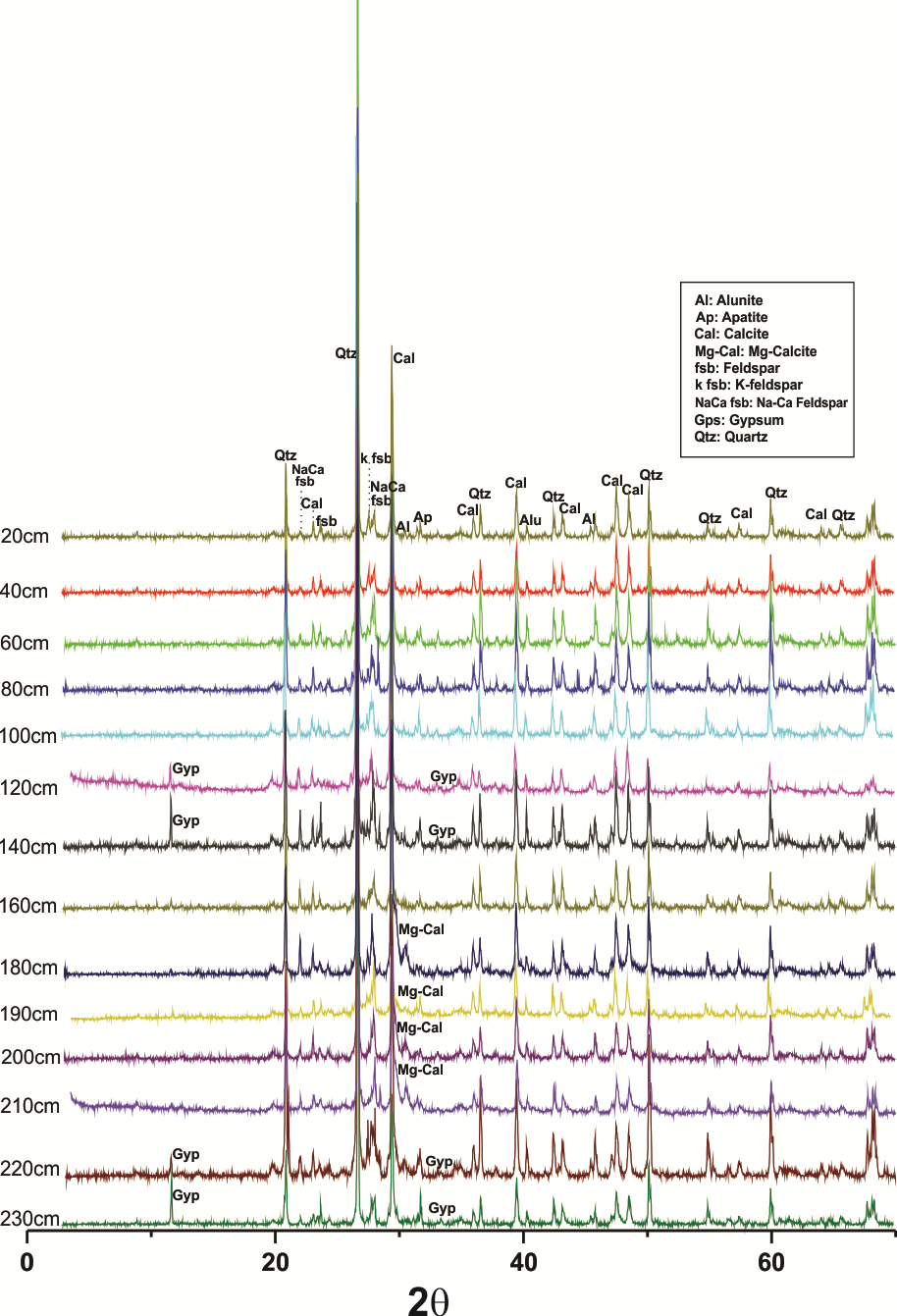


**Figure S5:** Mineralogical results from X-ray diffraction (XRD) analyses showing the presence of High-Mg calcite in core GeoB6201-5.


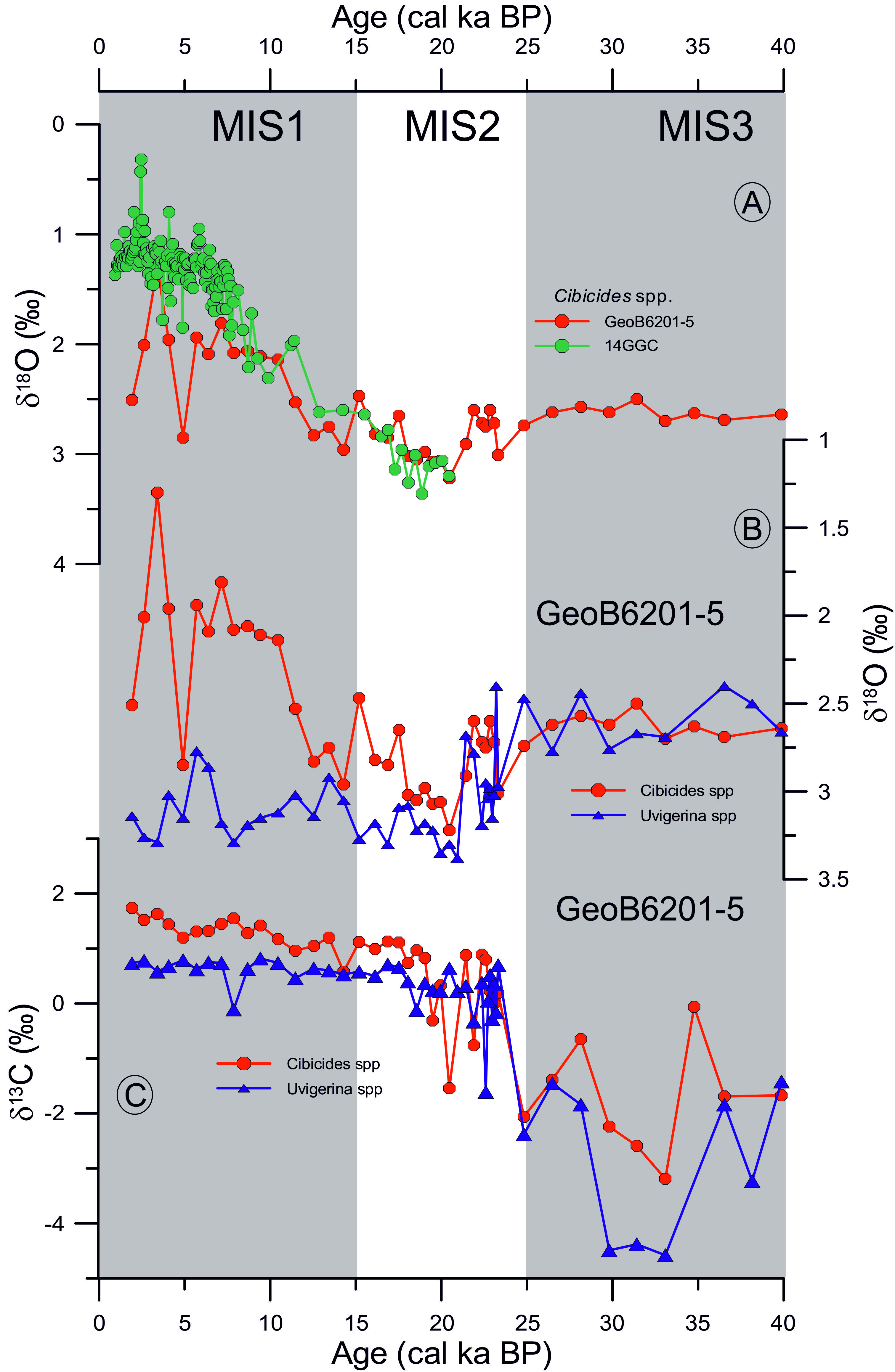


**Figure S6:** Benthic foraminifera stable isotope records over the last 40cal ka BP. (**A**) Comparison of *Cibicides* spp. δ^18^O records of the seep-core GeoB6201-5 and non-seep core 14GGC. (**B**) Comparison of δ^18^O between epfaunal (*Cibicides* spp) and infaunal (*Uvigerina* spp) taxa from core GeoB6201-5. In **B**, the δ^18^O values of *Uvigerina* spp were corrected by subtracting 0.47‰ following Marchito et al.^45^. (**C**) Comparison of benthic δ^13^C values from core GeoB6201-5.
